# Learning Curve of Cardiac Surgery Residents in Transit-Time Flow Measurement and High-Resolution Epicardial Ultrasonography During Coronary Surgery

**DOI:** 10.3390/jcm15020620

**Published:** 2026-01-13

**Authors:** Federico Cammertoni, Gabriele Di Giammarco, Nicola Testa, Natalia Pavone, Alberta Marcolini, Serena D’Avino, Piergiorgio Bruno, Maria Grandinetti, Francesco Bianchini, Antonio E. Trapani, Massimo Massetti

**Affiliations:** 1 Department of Cardiovascular Sciences, Fondazione Policlinico Universitario “A. Gemelli” IRCCS, 00168 Rome, Italy; nicola.testa@policlinicogemelli.it (N.T.); natalia.pavone@policlinicogemelli.it (N.P.); alberta.marcolini@guest.policlinicogemelli.it (A.M.); serenadavino93@gmail.com (S.D.); piergiorgio.bruno@policlinicogemelli.it (P.B.); maria.grandinetti@policlinicogemelli.it (M.G.); francesco.bianchini@guest.policlinicogemelli.it (F.B.); massimo.massetti@unicatt.it (M.M.); 2 Department of Neuroscience, Imaging and Clinical Science, School of Medicine and Health Science, Università “G. D’Annunzio” Chieti—Pescara, 66100 Chieti, Italy; gabrieledigiammarco57@gmail.com; 3 Faculty of Medicine and Surgery, Catholic University of Sacred Heart, 00168 Rome, Italy; aetrap99@gmail.com

**Keywords:** coronary artery bypass, cardiac surgical procedures/education, learning curve, intraoperative care/methods, ultrasonography/methods, physicians, residents/education

## Abstract

**Objectives**: This study aimed to define the learning curve required for cardiac surgery residents to acquire basic technical and interpretive skills in transit-time flow measurement (TTFM) and high-resolution epicardial ultrasonography (HRUS) during coronary artery bypass grafting (CABG). **Methods**: Prospective, observational, single-center study evaluating performance using a novel scoring system combining functional (TTFM) and anatomical (HRUS) assessment criteria. This study was registered on ClinicalTrials.gov (Identifier: NCT06589323). Nine cardiac surgery residents without prior hands-on experience in TTFM or HRUS were enrolled. Twenty-seven elective CABG patients (67 grafts) were analyzed. Each measurement was compared with those obtained by an expert benchmark surgeon (N.T.) under standardized hemodynamic conditions. **Results**: Residents achieved the predefined primary endpoint (combined TTFM + HRUS score/number of grafts ≥ 11) after a median of 3 cases (IQR 2–4) and 7 anastomoses (IQR 7–10). Kaplan–Meier analysis showed a progressive increase in the probability of success, with a sharp rise after the seventh anastomosis. A shorter interval between attempts (<30 days) was significantly associated with earlier achievement of the endpoint (*p* < 0.05). Median acquisition time for TTFM was 25 s, with <10% inter-observer variability across all flow parameters. HRUS images of adequate quality were obtained within 60 s in >90% of cases, though slightly lower success rates were observed for lateral and inferior wall targets. No resident- or procedure-related variable was independently associated with performance improvement. **Conclusions**: Mastery of basic TTFM and HRUS skills requires only a few cases and anastomoses, demonstrating a short and attainable learning curve. These findings challenge the perception of a steep learning process and support the routine use of intraoperative graft verification techniques in all CABG procedures.

## 1. Introduction

Early graft failure (<3 weeks) significantly increases morbidity and mortality in patients undergoing coronary artery bypass grafting (CABG) [1,2]. This adverse event occurs in approximately 4% of grafts [3] and is mainly related to technical factors, such as the quality of the anastomosis or the conduits used [4].

Transit-time flow measurement (TTFM) is an ultrasound-based technology that allows assessment of graft function while the patient is still in the operating room. In brief, a probe equipped with two transducers and an acoustic reflector generates ultrasound beams that propagate from the transducer to the reflector in both the upstream and downstream directions of blood flow. The blood volume flow is therefore derived from the difference in the transit times of these opposing acoustic beams. More specifically, this technique enables the acquisition of multiple parameters, which, when integrated and interpreted together, allow for assessment of the graft’s functional integrity. Although mostly based on retrospective studies, substantial evidence supports an association between abnormal flowmetry patterns and graft failure or major adverse clinical events [5,6].

High-resolution epicardial ultrasonography (HRUS) is an additional intraoperative imaging tool that complements flowmetry by providing real-time, direct imaging of the conduit, the anastomosis, and flow through them. When used together, TTFM and HRUS achieve high diagnostic accuracy and are essential for reducing the incidence of early graft failure [7].

Although the European Guidelines on Myocardial Revascularization [8] have been recommending the use of TTFM and HRUS during CABG procedures, their application remains unjustifiably limited. Indeed, among more than 800,000 CABG procedures performed in 2017, only 30% included intraoperative quality control with TTFM [9].

A steep learning curve in acquiring and interpreting data from TTFM and HRUS has been suggested as one of the possible reasons for their underutilization [10]. Conversely, advocates of these techniques argue that the learning curve is relatively short. Specifically, proficiency in the basic principles of TTFM and HRUS is reportedly achieved after approximately 10 to 20 cases [4,9]. However, these estimates are empirical, and no study has objectively quantified the complexity of the training process for these modalities.

Therefore, the aim of this prospective, observational, single-center study is to describe the learning curve for basic TTFM and HRUS skills in a cohort of cardiac surgery residents with no prior practical experience using these techniques.

## 2. Materials and Methods

### 2.1. Study Population

**Figure 1:**
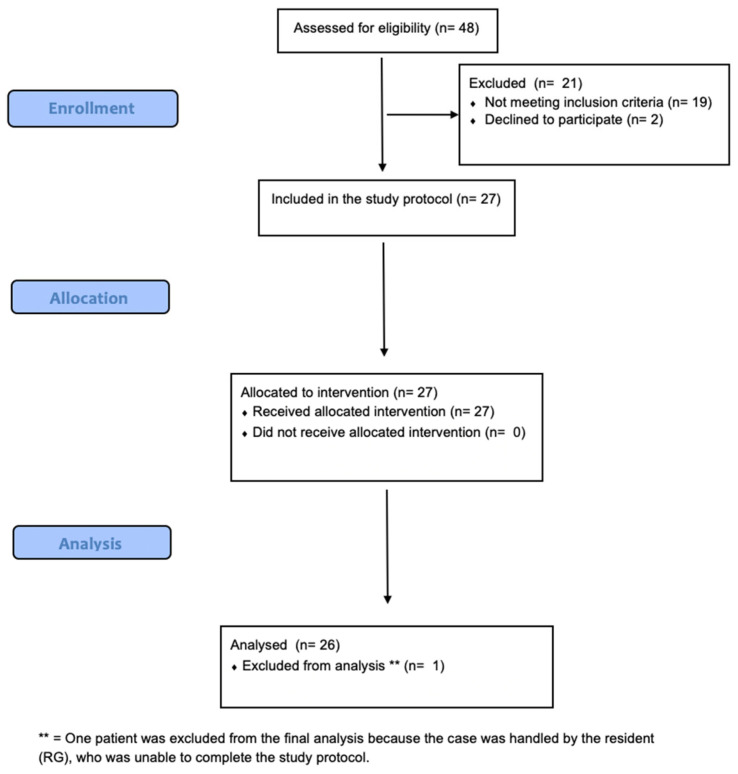
**Flow diagram describing the process of patient selection, allocation and data analysis**.

Adult patients (≥18 years) scheduled for CABG were eligible for this study. Those undergoing emergency/urgent procedures or combined cardiac surgeries were excluded. As well, patients who underwent off-pump CABG were excluded, as in these cases epicardial imaging requires a higher level of operator expertise [4,9], and its assessment falls outside the scope of this study, which is focused on basic skill acquisition. For the same reason, patients undergoing sequential grafting or using Y-graft configurations were not included in the analysis. In Figure 1, the flow diagram describes the process of patient enrollment, allocation and data analysis.

All enrolled patients provided written informed consent to participate in the clinical investigation.

### 2.2. Cardiac Surgery Residents

In September 2024, nine cardiac surgery residents from our institution were enrolled in the protocol. Particularly, the cohort included two first-year residents (RP, GM), one second-year resident (KC), three third-year residents (ED, MM, and AO), two fourth-year residents (GC, RG), and one fifth-year resident (SD).

Due to relocation to another hospital, one participant (RG, fourth-year resident) was unable to complete the study. Consequently, the learning curves of eight residents were analyzed and reported.

Both TTFM and HRUS are routinely employed in our institution. However, although all residents were familiar with these modalities, none had prior hands-on experience with their use. In standard practice, the acquisition of flow measurements and epicardial imaging is performed by the operating or assistant surgeon.

Certainly, residents’ baseline technical knowledge and skills, including their understanding of TTFM and HRUS, varied according to their year of training, and this was considered in the data analysis.

All participants provided written informed consent to take part in the study. To avoid any potential coercion, study information and consent collection were conducted by a physician other than the residents’ supervising tutor.

### 2.3. Pre-Training and Benchmark Selection

Before the start of the study, a senior cardiac surgeon (NT) with extensive experience in the use of TTFM and HRUS was designated as the benchmark operator for the residents’ performance assessment. The study protocol was applied exclusively to patients operated on by this benchmark surgeon. This approach ensured a standardized evaluation of resident performance but consequently prolonged the enrollment period.

**Figure 2:**
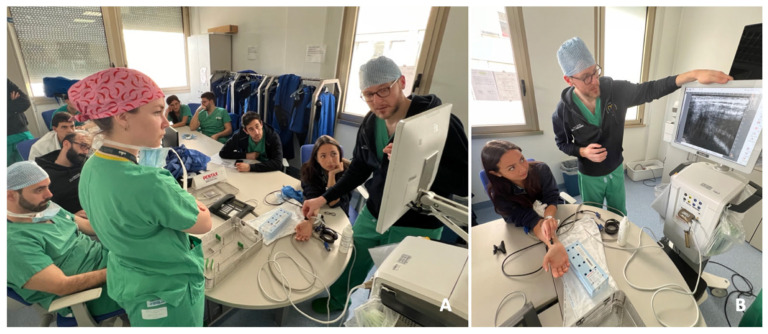
**Cardiac surgery residents’ training:** (**A**) Before the start of the study, all residents received a theoretical and practical training session on transit-time flow measurement and high-resolution epicardial ultrasonography, conducted by the benchmark surgeon. (**B**) In addition, residents were given the opportunity for an additional hands-on session, during which they practiced epicardial ultrasonography on their own radial artery.

Prior to study initiation, the supervisor conducted a comprehensive briefing covering both the theoretical and practical aspects of TTFM and HRUS. Residents were then given the opportunity to familiarize themselves with the HRUS device through an additional hands-on session, during which they practiced imaging of their own radial artery (Figure 2). For this study, the MiraQ System (Medistim AS, Oslo, Norway) was used.

### 2.4. HRUS Study Protocol

Upon completion of each distal anastomosis, the resident performed both a short-axis and a long-axis projection using 2D imaging and color-flow mapping. A time limit of one minute was set for each projection, based on previously published experience [11,12].

**Figure 3:**
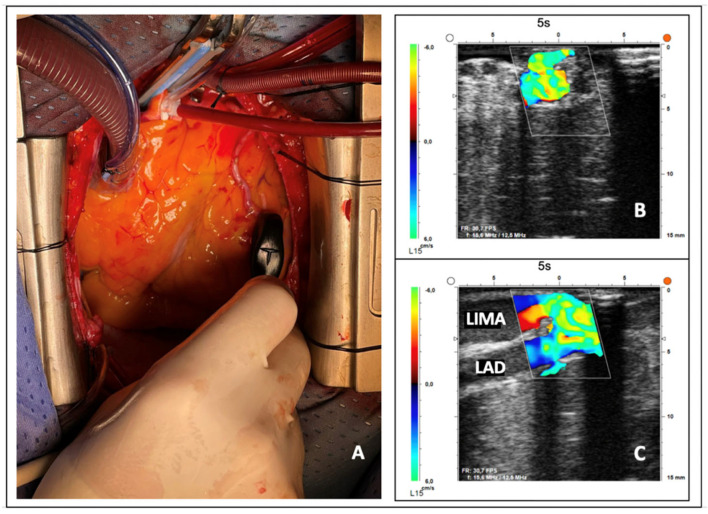
**High-resolution epicardial ultrasound (HRUS) scoring system.** After completion of each distal anastomosis, the resident performed HRUS acquisition (**A**). The objective was to obtain, within a 1−min time limit, both a short-axis (**B**) and a long-axis (**C**) 2D and color-flow image. The scoring system followed these criteria: short−axis acquisition within 60 s = 1 point; adequate short-axis color−flow acquisition = 1 point; long-axis acquisition within 60 s = 1 point; adequate long-axis color-flow acquisition = 1 point. The maximum achievable score was 4, and the minimum was 0. After image acquisition, the resident was required to assess the quality of the anastomosis. In the event of a misjudgment (i.e., a patent anastomosis incorrectly classified as inadequate, or vice versa), the total score for that graft (HRUS + TTFM) was reset to zero. A projection was considered adequate when the following criteria were met: for the short−axis view, perpendicular alignment to the graft axis with clear visualization of the lateral borders, central lumen, and posterior wall of the anastomosis; for the long−axis view, correct longitudinal alignment showing the heel, toe, and receiving coronary vessel, both proximal and distal to the anastomosis. **Abbreviations:** LIMA = left internal mammary artery; LAD = left anterior descending artery.

Subsequently, the benchmark surgeon conducted an independent evaluation of the same anastomosis. The HRUS scoring system and the parameters used to assess image quality are detailed in Figure 3. The maximum achievable score was 4, and the minimum was 0. Regardless of the numerical score obtained, if the resident misjudged the technical adequacy of the anastomosis—that is, if an imperfect anastomosis was considered correct or a proper one was judged defective—the total score for that graft (TTFM score + HRUS score) was reset to zero. Assessment of each resident’s performance was carried out postoperatively by reviewing the recorded ultrasound videos stored on the MiraQ device. Proximal anastomoses, which were assessed intraoperatively only by the benchmark surgeon, were not included in the analysis.

### 2.5. TTFM Study Protocol

TTFM measurements were obtained at the end of cardiopulmonary bypass, immediately before protamine administration. Specifically, each graft was assessed first by the resident and then by the benchmark surgeon, ensuring that the mean arterial pressure (MAP) was >70 mmHg, comparable between the two measurements (pressure difference < 10%), and that no inotropic or vasoconstrictive drugs were started between the two acquisitions. A time limit of one minute was established for each measurement. The following parameters were recorded: mean graft flow (MGF), pulsatility index (PI), percentage of backward flow (%BF), and percentage of diastolic filling (%DF). In addition, the selected probe size, the need for repeated measurements, and the quality of the acoustic coupling index (ACI) were documented.

**Figure 4:**
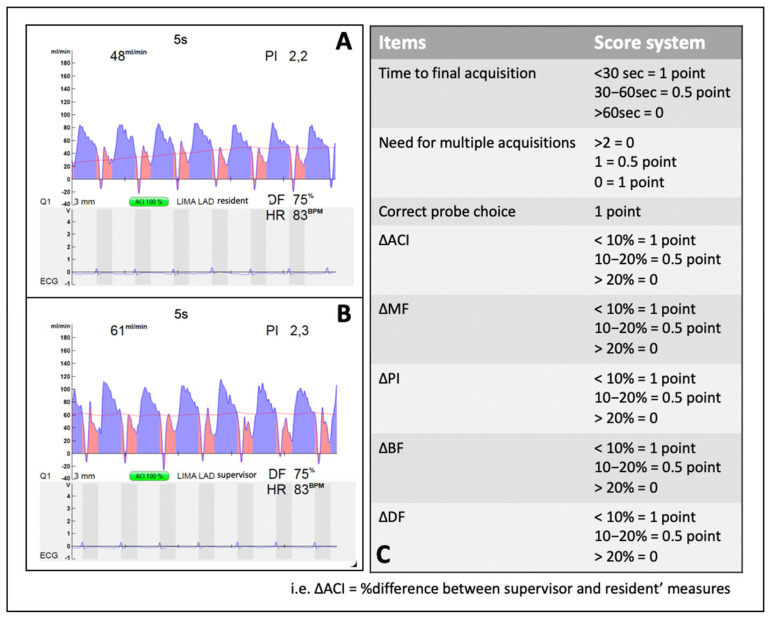
**Transit-time flow measurement (TTFM) scoring system.** TTFM measurements were obtained at the end of cardiopulmonary bypass, before protamine administration. The resident (**A**) performed the measurement immediately before the benchmark surgeon (**B**). Both measurements were acquired under comparable hemodynamic conditions, ensuring a mean arterial pressure > 70 mmHg, a pressure difference (Δ) < 10%, and no infusion of vasoactive drugs between recordings. The scoring system, illustrated in panel (**C**), accounted for the following items: time required for measurement acquisition; ability to select the appropriately sized probe; ability to avoid repeated measurements; percentage difference (%Δ) in the main flow parameters: ACI = acoustic coupling index; MF = mean flow; PI = pulsatility index; DF = diastolic filling; %BF = backward flow. The maximum achievable score was 8, and the minimum was 0. At the end of each TTFM measurement, the resident was required to judge graft quality. In the event of a misjudgment (i.e., a graft incorrectly classified as patent), the total graft score (combined HRUS + TTFM) was reset to zero.

The scoring system, summarized in Figure 4, was based on the percentage difference (%Δ) between the resident’s and the benchmark surgeon’s measurements. The maximum achievable score was 8, and the minimum was 0. As with the HRUS evaluation, after completing the flow measurement, the resident was required to assess graft quality subjectively. If this assessment was incorrect—that is, if a compromised graft was judged satisfactory (or vice versa)—the total score for that graft (TTFM score + HRUS score) was reset to zero.

### 2.6. Data Management

For each case, individual TTFM and HRUS reports were stored on the device and also exported and archived in a dedicated electronic folder. Data obtained from both the resident and the benchmark surgeon were entered into a dedicated database, together with relevant patient and operative variables (patient age and sex, date of surgery, total number of bypass grafts performed, number of arterial and venous conduits used, conduit configuration strategy, total operative time, duration of cardiopulmonary bypass, and aortic cross-clamp time).

### 2.7. Endpoints

The learning curve was considered complete upon achievement of a predefined primary endpoint. Specifically, residents were required to obtain, in a single case, a combined HRUS + TTFM score divided by the number of grafts analyzed ≥ 11. This cutoff was chosen because it represents 90% of the maximum achievable score. Since this is a study on the acquisition of fundamental competencies, we considered that the endpoint should be placed as high as possible. For this reason, we excluded the option of setting it at lower thresholds (80% of the maximum value, etc.), which would have represented an unjustifiable compromise. It must be noted that the design of the scoring system and the specific target value for the primary endpoint were original to this study, as no prior data in the literature were available to guide the selection of an alternative scoring approach or cutoff value. Moreover, it is important to note from the outset that while the TTFM score is based on strictly objective criteria (i.e., differences between the trainee’s and the benchmark surgeon’s flowmetric measurements), the HRUS score also relies on a qualitative—and non-blinded—assessment by the senior surgeon (appropriateness of short- and long-axis acquisitions). However, this evaluation pertains to predefined and well-specified parameters (Figure 3), thereby minimizing the potential introduction of biases.

Secondary endpoints included the following:

- A TTFM total score per number of grafts analyzed ≥ 7;
- An HRUS total score per number of grafts analyzed ≥ 4.

This clinical study was approved by the local Ethics Committee (protocol No. 0021423/24, dated 3 September 2024) and registered on ClinicalTrials.gov (Identifier: NCT06589323).

### 2.8. Statistical Analysis

Continuous variables are shown as mean ± standard deviation if normally distributed and as median (first-third quartile) otherwise. Percentages are used to describe categorical variables. The Kolmogorov–Smirnov test was used to check the normality/skewness of continuous variables before further analysis. Groups were compared using Fisher’s exact test or λ^2^ test for categorical variables, as appropriate. Instead, continuous variables were compared using an independent-samples *t*-test or Mann–Whitney U tests, as appropriate. The Pearson λ^2^ test and the Kruskal–Wallis H test were used to compare categorical variables or non-normally distributed continuous variables, respectively, across multiple independent groups. All tests were two-sided, and a type I error significance level of 0.05 was considered. The Kaplan–Meier method was employed to estimate the cumulative probability of achieving the primary endpoint, while subgroup differences were assessed using the log-rank test. A multiple logistic regression analysis using an “enter” method was performed to identify a relationship between resident-related (year of residency, dominant hand, previous echo experience) or procedure-related (time interval between procedures, myocardial wall site) variables and the primary endpoint achievement.

Mixed-effect models were not applied because the unit of analysis was the resident-level attempt, with very few observations per cluster and limited within-cluster variability, conditions that would have produced unstable or non-identifiable random-effect estimates. Statistical analysis was performed with MedCalc v. 22.0 (MedCalc Software Ltd., Ostend, Belgium).

## 3. Results

### 3.1. Baseline Patient Characteristics and Surgery Details

From September 2024 to March 2025, a total of 27 patients were enrolled in the study. Their baseline characteristics are summarized in Table 1.

The mean age was 65.6 ± 7.4 years, and the majority were male (92.6%), with a high prevalence of cardiovascular risk factors. Most patients were asymptomatic (44.4%), had preserved left ventricular function, and were classified as low surgical risk. One patient was excluded from the final analysis because the case was handled by the resident (RG), who was unable to complete the study protocol.

In total, 67 grafts were performed and analyzed (Table 2): 28 arterial (41.8%) and 39 venous (58.2%). In no case was revision of an anastomosis required based on TTFM or HRUS findings.

The left internal mammary artery “in situ” was the most commonly used conduit for revascularization of the left anterior descending artery (96.2%). Other coronary targets were most frequently revascularized using saphenous vein grafts. Exceptions included the use of the “in situ” right internal mammary artery in two patients, and the radial artery in one patient, to graft the intermediate branch and the first obtuse marginal branch, respectively. The mean total operative time, cardiopulmonary bypass time, and aortic cross-clamp time were 261.5 ± 42.0, 82.6 ± 23.4, and 65.2 ± 19.5 min, respectively.

### 3.2. Primary Endpoint Analysis

**Figure 5:**
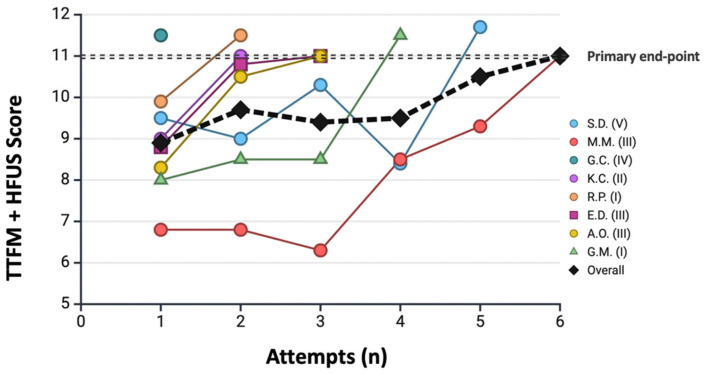
**Progression toward the primary learning endpoint across consecutive attempts.** Each resident is represented by a different color/symbol, with the corresponding year of training indicated in the legend. The black line represents the cumulative curve. The primary endpoint (dotted line) is defined as achieving a combined HRUS + TTFM total score divided by the number of analyzed grafts ≥ 11. **Abbreviations:** HRUS = high-resolution epicardial ultrasonography; TTFM = transit-time flow measurement.

A median of 3 cases (IQR 2–4) and 7 anastomoses (IQR 7–10) were required to achieve the primary endpoint. Detailed results for each resident are reported in Table 3, and the corresponding learning curve is shown in Figure 5.

**Figure 6:**
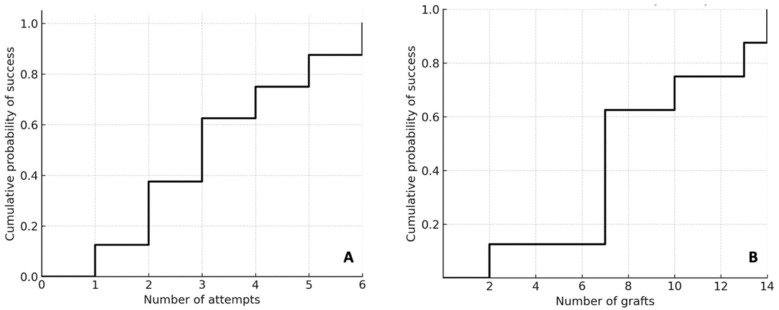
**Kaplan–Meier analysis.** These curves illustrate the cumulative probability of achieving the primary endpoint as a function of the number of attempts (**A**) and the number of analyzed anastomoses (**B**).

**Figure 7:**
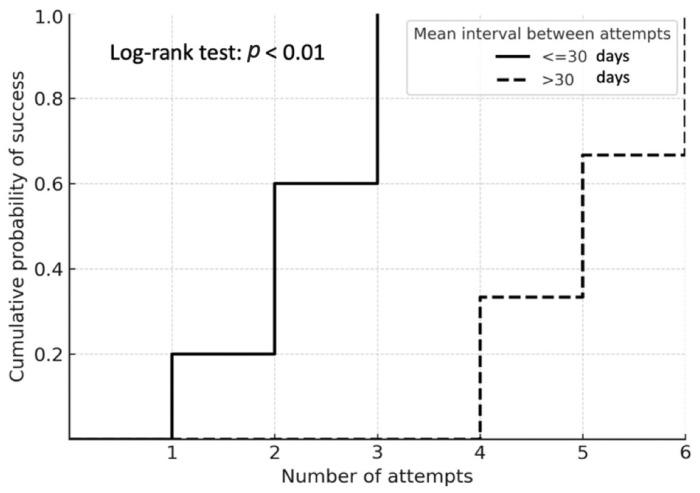
**Kaplan–Meier analysis.** These curves illustrate the cumulative probability of achieving the primary endpoint, comparing residents with a mean interval between attempts of >30 days versus <30 days. The log-rank test indicates a statistically significant difference between the two subgroups.

The Kaplan–Meier analysis demonstrated that the probability of reaching the primary endpoint increased progressively with the number of analyzed anastomoses, with a notable inflection after the seventh anastomosis (Figure 6). The median interval between attempts was 36 days (IQR 23–67). When stratified by this variable, residents with a median inter-attempt interval <30 days reached the primary endpoint significantly earlier than those with intervals >30 days (Figure 7).

### 3.3. Secondary Endpoint Analysis

To achieve the secondary endpoint related to TTFM, a median of 1.5 cases (IQR 1.0–3.2) and 5.5 analyzed anastomoses (IQR 2.7–7.7) were required. The results of the TTFM analysis, stratified by coronary target, are reported in Table 4.

**Figure 8:**
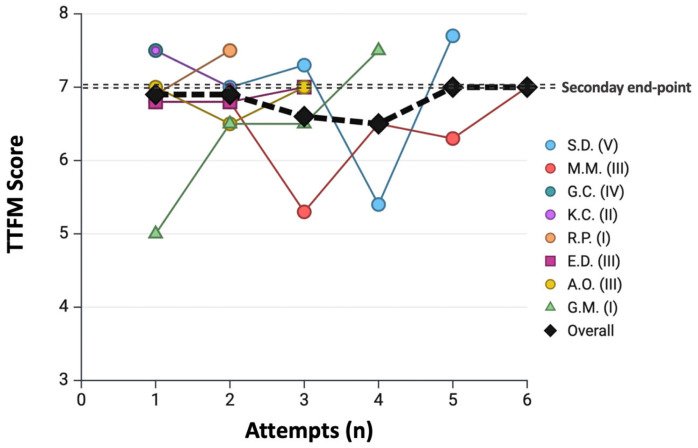
**Learning curve for transit-time flow measurement performance.** Each resident is represented by a different color/symbol, with the corresponding year of training indicated in the legend. The black line represents the cumulative curve. The secondary endpoint related to TTFM (dotted line) is defined as achieving a total TTFM score divided by the number of analyzed grafts ≥ 7.

The median acquisition time was 25 s (IQR 21–35). In 6 cases (8.9%), multiple acquisitions were required before obtaining the final measurement, and in 2 cases (3%), a change in probe size was necessary. The percentage difference between residents’ and benchmark surgeon’s flow parameters was globally low and consistently < 10%: ΔACI%: 7.4% (3.4–12.8); ΔMF%: 7.5% (4.3–14.8); ΔPI%: 7.7% (4.5–16.5); ΔDF%: 3.0% (1.5–7.0); ΔBF%: 4.9% (2.4–8.3). No statistically significant differences were observed between coronary territories for any of the analyzed variables. In all cases, residents correctly interpreted the flow parameters. The TTFM learning curve is summarized in Figure 8.

To achieve the secondary endpoint related to HRUS, a median of 2 cases (IQR 2.0–4.2) and 7 analyzed anastomoses (IQR 4.0–10.7) were required. Short-axis images were acquired within 60 s in 94% of cases (63/67), and adequate image quality (as judged by the benchmark surgeon) was achieved in 61% (41/67). Long-axis images were acquired within 60 s in 95% of cases (64/67) and were considered adequate in 71.6% (48/67) (Table 5).

**Figure 9:**
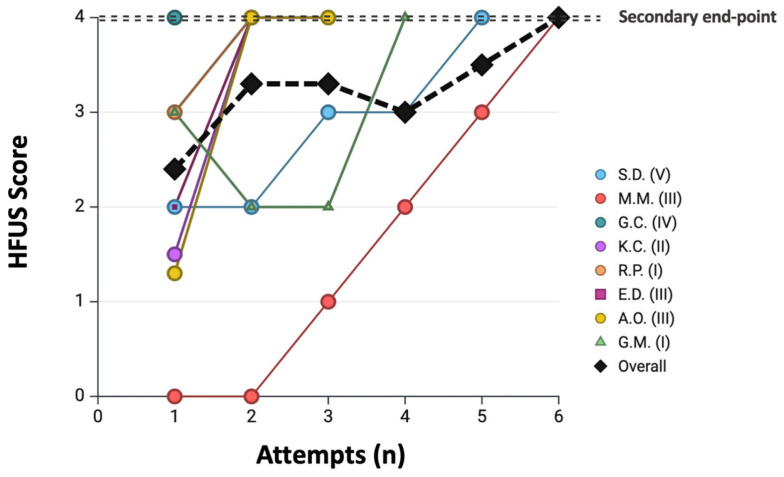
**Learning curve for high-resolution epicardial ultrasound performance.** Each resident is represented by a different color, with the corresponding year of training indicated in the legend. The black line represents the cumulative curve. The secondary endpoint related to HRUS (dotted line) is defined as achieving a total HRUS score divided by the number of analyzed grafts ≥ 4.

Notably, adequate-quality imaging in both short- and long-axis projections was obtained in 73.1% of grafts on the left anterior descending artery, compared with 50.0%, 35.3%, and 35.7% for the anterolateral, lateral, and inferior walls, respectively (*p* = 0.04). In all cases, residents accurately assessed the quality of the anastomoses. The HRUS learning curve is illustrated in Figure 9.

### 3.4. Multiple Logistic Regression Analysis

The multivariable logistic regression analysis is reported in Table 6. Neither resident-related factors (year of training, dominant hand, prior ultrasound experience) nor procedure-related factors (acquisitions on the lateral or inferior ventricular wall) were significantly associated with the achievement of the primary endpoint.

## 4. Discussion

Although coronary angiography remains the gold standard for assessing graft quality, its logistical limitations (need for additional equipment and personnel, higher costs, and longer operative time) and clinical drawbacks (invasiveness and use of contrast agents) restrict its use to selected cases [10,13]. Nevertheless, verifying graft patency before leaving the operating room is essential to minimize the risk of adverse postoperative events [1,2] and should ideally be performed systematically.

For many years—and still in common practice—the coronary surgeon has relied on electrocardiographic stability, hemodynamic parameters, and regional wall motion assessment by transesophageal echocardiography to infer the adequacy of myocardial revascularization. However, these indicators cannot reliably confirm graft function. In a non-negligible proportion of cases, a graft may be occluded without any clinical or hemodynamic evidence [14]. Moreover, technical issues at the time of surgery may result in delayed graft failure, manifesting hours or even days after the procedure.

Over the past three decades, several intraoperative modalities have been proposed to verify graft patency. Among these, transit-time flow measurement has stood the test of time as the simplest, most cost-effective, reproducible, and minimally invasive method currently available. First reported by Walpoth et al. for the intraoperative detection of graft failure [15], TTFM use has since been associated with reduced postoperative adverse events and mortality after CABG [14,16]. Laali et al. reported the outcomes of 430 patients in whom TTFM was used compared with 480 patients in whom this technique was not employed. Although the TTFM group had slightly longer cardiopulmonary bypass times, the incidence of MACE was significantly lower (3.3% vs. 6.9%). Of note, TTFM was associated with a 54% reduction in the risk of MACE [17].

TTFM demonstrates high specificity, ranging from 94% to 98%, and a lower sensitivity of 25–30% [7]. In approximately 10–15% of cases, TTFM interpretation may be technically challenging or frankly equivocal [9]. In a seminal paper, Thuijs and colleagues reported that TTFM could lead to graft revision in up to 4.3% of patients undergoing CABG, with a pooled rate of revisions among abnormal grafts of 25.1% [18].

Following numerous preliminary experiences and substantial technical improvements [11,12,19,20], HRUS now allows the addition of an anatomical assessment of both proximal and distal anastomoses, as well as of the grafts themselves, to the functional evaluation provided by TTFM. Reported sensitivity and specificity of HRUS are 98% and 100%, respectively [21].

Moreover, HRUS can be employed to identify the optimal site for constructing the distal anastomosis, to detect intramyocardial coronary segments, and to recognize atherosclerotic plaques in the ascending aorta [9]. Di Giammarco et al. demonstrated that the addition of HRUS to TTFM increases the positive predictive value of intraoperative graft assessment, with the combination of the two modalities approaching 100% diagnostic accuracy [7].

In the REQUEST study [22], the combined use of TTFM and HRUS enabled identification of issues involving the aorta, in situ conduits, coronary targets, or anastomoses, leading to a change in surgical strategy in 25.2% of patients.

The latest European Guidelines on Myocardial Revascularization recommend the systematic use of these modalities during CABG—not only in suspected cases but in all procedures [8].

Despite these recommendations, in 2017 only 30% of the approximately 800,000 CABG procedures performed worldwide were assessed for graft patency using TTFM [8], with utilization rates varying widely between centers—from <1% to over 80% [9]. More recently, the REQUEST study also reported an unexpectedly low use of HRUS, even though its employment was explicitly included in the study protocol [22]. Internal data published in 2023 by the manufacturers of flowmetry and high-resolution epicardial ultrasound devices indicate that fewer than 35% of CABG procedures performed in the United States, and approximately 40% of those performed in Canada and Europe (with substantial variability between countries), undergo intraoperative quality assessment using these techniques [10].

The reasons behind this underutilization are multifactorial and may include doubts about their diagnostic accuracy, high costs, complexity of their integration in the surgical workflow (with longer operative times), and skepticism about clinical utility [10]. Not least, concerns about a steep learning curve and the perceived need for specific training have been advocated [10]. However, according to some authors, acquisition of the basic TTFM skills may require as few as 10–15 cases, whereas HRUS appears to have a slightly more challenging learning curve, requiring at least 20 cases [4,9]. Notably, in the REQUEST study, participation required surgeons to have previously performed a minimum of 20 cases using both TTFM and HRUS [22].

Undeniably, mastering these intraoperative imaging techniques requires experience and repeated measurements. The aim of our study, however, was to objectively assess the acquisition of basic competencies—that is, the ability to correctly obtain and interpret both a flowmetry tracing and an epicardial ultrasound image. For this purpose, we designed a novel, dedicated scoring system for TTFM and HRUS, incorporating the key technical and interpretative items of both modalities.

Importantly, this scoring system accounted not only for the acquisition aspect (e.g., the percentage difference in flow parameters compared with the benchmark surgeon’s measurement) but also for the interpretative component—operationalized as the residents’ ability to correctly judge graft adequacy in cases with normal TTFM and HRUS findings.

Each resident’s measurement was directly compared with that of the same experienced benchmark surgeon for all cases. Although this approach prolonged the duration of the study and increased the interval between attempts, it ensured standardized evaluation and minimized potential detection bias.

We deliberately designed a controlled learning environment for TTFM and HRUS training, selecting elective, low-risk CABG patients predominantly undergoing mixed revascularization strategies. This approach provided a safe and stable setting for all parties involved—the patient, the surgical team, and the resident. Certainly, this protected environment does not reflect real-world clinical practice, where one may encounter unstable patients, urgent procedures, or grafts with complex configurations. While this limits the generalizability of our findings, it is nonetheless a necessary prerequisite for objectively assessing the learning curve related to the basic TTFM and HRUS skills.

The most notable finding of our study is that the learning curve for both TTFM and HRUS was remarkably short, requiring a median of 3 cases and 7 analyzed anastomoses to reach the primary endpoint. Specifically, TTFM learning proved to be straightforward: residents demonstrated early confidence in probe selection, acquired measurements rapidly (mean < 30 s), and maintained a percentage error < 10% across all parameters, with no differences among graft types or coronary territories.

HRUS acquisition was also rapid, though slightly more challenging in certain myocardial territories where residents found it more difficult to obtain adequate-quality images. This was likely due to technical challenges in orienting the probe when the heart was displaced to expose the lateral or inferior walls. Nevertheless, after only a few attempts, all residents demonstrated the ability to effectively handle and orient the probe. This observation aligns with previous findings by Taggart et al. [22].

The wide variability in the time intervals between successive attempts among residents further revealed that those with shorter intervals were significantly more likely to achieve the primary endpoint earlier. No other factors—such as year of training, dominant hand, or previous echocardiography experience—were associated with improved performance or faster progression along the learning curve.

### Study Limitations

This study presents several limitations.

First, all grafts analyzed in this series demonstrated normal TTFM and HRUS findings. While this is clinically reassuring, it prevents us from determining whether residents were also capable of recognizing abnormal or pathological flow and imaging patterns. Given the low probability of encountering a dysfunctional graft (center-dependent but generally < 5%), a study designed in a completely different manner would have been required to selectively investigate what happens when a trainee is confronted with such a scenario. In other words, it would have been necessary to specifically examine the learning curve for dysfunctional grafts. The present study, instead, was conceived to assess the preceding step—namely, the ability to acquire and interpret normal TTFM and HRUS findings. Naturally, we had to take into account the possibility of encountering pathological grafts, which is why the scoring system includes an interpretative component. However, the reader should be aware that the interpretative ability to recognize an abnormal graft (namely, a graft that must be revised) has not been tested.

Second, the HRUS scoring system was based on a qualitative assessment of the images acquired by the residents. Although the evaluator was always the same benchmark surgeon, and predefined parameters for adequacy were established before study initiation, the possibility of observer bias in score attribution cannot be excluded. Another limitation concerns the small number of patients and analyzed anastomoses required for all residents to reach the primary endpoint, which was lower than initially expected. This inherently limits the statistical power of our analysis. In particular, the multivariable regression analysis is underpowered, and results should be interpreted with caution. In addition, some may argue that the overall duration of the study and the interval between successive attempts were unacceptably long. However, this design choice was intentional, aiming to avoid any compromise in patient care. Moreover, the time elapsed between sessions likely allowed residents to mentally review, absorb, and consolidate the procedural steps more effectively than if measurements had been performed in rapid or consecutive succession. Finally, we excluded complex graft configurations (such as sequential or Y-shaped grafts) and did not include proximal anastomoses, as our goal was to evaluate basic technical competencies in standard settings. Further studies will be required to explore learning dynamics in more complex graft configurations.

## 5. Conclusions

This study investigated the learning curve complexity associated with acquiring basic skills in transit-time flow measurement and high-resolution epicardial ultrasonography among cardiac surgery residents.

Our findings suggest that, at least for basic acquisition and interpretation of normal intraoperative findings in elective, on-pump CABG, the learning curve is short. Larger, multicenter studies including more complex graft configurations and abnormal cases are needed before definitively ruling out the learning curve as a barrier to adoption.

If these conclusions are confirmed, the argument of a supposedly steep learning curve for TTFM and HRUS could no longer be used to justify the lack of adoption of these techniques in coronary surgery.

## Figures and Tables

**Table 1:** Baseline characteristics of the 27 patients enrolled in the LEARNERS study.

Variable	
Age, years	65.6 ± 7.4
Male	25 (92.6)
BMI, Kg/m^2^	27.5 ± 4.8
Hypertension	20 (74.1)
Dyslipidemia	25 (92.6)
Diabetes	12 (44.4)
Active smoker	9 (33.3)
COPD	5 (18.5)
Previous PCI	5 (18.5)
Clinical presentation	
Asymptomatic	12 (44.4)
Stable angina	5 (18.5)
Unstable angina	6 (22.2)
NSTEMI	4 (14.8)
PAD	10 (37)
Previous stroke	4 (14.8)
eGFR, mL/min	87.3 ± 17.4
Preoperative dialysis	0 (0)
EF, %	57.0 ± 6.5
STS Score	0.9 ± 0.7

**Abbreviations.** BMI = body mass index; COPD = chronic obstructive pulmonary disease; EF = ejection fraction; eGFR = estimated glomerular filtration rate (Cockcroft and Gault formula); NSTEMI = non-ST-segment elevation myocardial infarction; PAD = peripheral artery disease; PCI = Percutaneous Coronary Intervention; STS Score = Society of Thoracic Surgeons score.

**Table 2:** Type and distribution of the coronary artery bypass grafts performed in the 26 LEARNERS study patients.

Variable	
**Total grafts**	67
Graft per patient	2.6 ± 0.6
	
**Arterial graft**	28 (41.8)
Arterial graft per patient	1.1 ± 0.4
	
**Venous graft**	39 (58.2)
Venous graft per patient	1.5 ± 0.9
**Coronary target and graft type:**	
**LAD**	26
LIMA	25 (96.2)
SVG	1 (3.8)
**Diagonal branches**	8
SVG	8 (100)
**OM**	17
SVG	15 (88.2)
“in situ” RIMA	1 (5.9)
“free graft” RA	1 (5.9)
**Ramus**	2
SVG	1 (50)
“in situ” RIMA	1 (50)
**RCA**	1
SVG	1 (100)
**PDA**	13
SVG	13 (100)
Surgery time, min	261.5 ± 42.0
X-clamp time, min	65.2 ± 19.5
ECC time, min	82.6 ± 23.4

**Abbreviations:** ECC = extracorporeal circulation; LAD = left anterior descending artery; LIMA = left internal mammary artery; OM = obtuse marginal branches; PDA = posterior descending artery; RA = radial artery; RCA = right coronary artery; RIMA = right internal mammary artery; X-clamp = cross-clamping; SVG = saphenous vein graft.

**Table 3:** Number of attempts/grafts needed and median time intervals for each resident to meet the primary endpoint.

Resident	PG Year	n° of Attempts	n° of Grafts	Δt, days
S.D.	5°	5	14	66 (60–72)
G.C.	4°	1	2	-
M.M.	3°	6	13	35 (28–94)
E.D.	3°	3	7	29 (22–35)
A.O.	3°	3	7	17 (12–22)
K.C.	2°	2	7	5 (-)
G.M.	1°	4	10	36 (31–81)
R.P.	1°	2	7	1 (-)
**Overall**		**26**	**67**	**36 (23–67)**

**Abbreviations.** PG = post-graduate year.

**Table 4:** Transit-time flowmetry results stratified by coronary territory among the 26 patients in the LEARNEARS study.

TTFM	LAD (*n* = 26)	Diag/Ramus (*n* = 10)	OM (*n* = 17)	RCA/PDA (*n* = 14)	*p*
Acquisition time, s	26 (25–35)	24 (17.0–30.8)	24.5 (21.0–29.8)	26.0 (18.5–36.5)	0.42
Multiple attempts	3 (11.5)	0 (0)	1 (5.9)	2 (14.3)	0.61
Probe change	0 (0)	0 (0)	0 (0)	2 (14.3)	1
ΔACI, %	7.1 (4.5–11.4)	9.2 (5.3–15.8)	7.3 (3.4–12.8)	4.9 (3.0–16.2)	0.88
ΔMF, %	9.6 (5.0–22.0)	8.0 (6.0–14.7)	6.8 (4.2–13.7)	5.7 (1.9–8.6)	0.22
ΔPI, %	6.4 (4.3–14.2)	6.0 (3.0–11.2)	7.4 (5.1–15.8)	12.5 (9.5–38.1)	0.10
ΔDF, %	2.6 (1.5–8.2)	5.6 (3.3–7.2)	3.7 (1.2–9.8)	2.9 (1.5–3.0)	0.41
ΔBF, %	4.6 (2.3–7.9)	5.3 (2.5–8.4)	4.9 (2.1–8.2)	5.1 (2.7–8.5)	0.37

ΔACI, ΔMF, ΔPI, ΔDF, and ΔBF denote the percentage differences between the measurements obtained by the resident and those recorded by the benchmark surgeon (NT) for ACI, MF, PI, DF, and BF, respectively. **Abbreviations:** ACI = acoustic coupling index; MF = mean flow; PI = pulsatility index; DF = diastolic filling; BF = backward flow; LAD = left anterior descending artery; Diag = diagonal artery; OM = obtuse marginal; RCA/PDA = right coronary artery/posterior descending artery.

**Table 5:** HRUS results stratified by coronary territory among the 26 patients in the LEARNEARS study.

HRUS	LAD (26)	OM (17)	Diag/Ramus (10)	RCA/PDA (14)	*p*
Acquisition time < 60 s					
Short ax.	25 (96.2)	16 (94.1)	10 (100)	12 (85.7)	0.46
Long ax.	26 (100)	15 (88.2)	10 (100)	13 (92.9)	0.25
Both	25 (96.2)	15 (88.2)	10 (100)	12 (85.7)	0.44
**Appropriate acquisition**					
Short ax.	20 (76.9)	10 (58.8)	5 (50)	6 (42.9)	0.15
Long ax.	22 (84.6)	10 (58.8)	6 (60)	10 (71.4)	0.24
Both	19 (73.1)	6 (35.3)	5 (50)	5 (35.7)	0.04

**Abbreviations:** LAD = left anterior descending artery; Diag = diagonal artery; OM = obtuse marginal; RCA/PDA = right coronary artery/posterior descending artery.

**Table 6:** Multiple logistic regression analysis for the primary endpoint.

	Odds Ratio	Standard Error	95% CI	*p*-Value
**Resident-related**				
PGY > 3	0.89	0.14	0.67–1.17	0.38
Previous echo expertise	1.49	0.46	0.61–3.68	0.40
Ambidextrous	0.84	0.31	0.45–1.63	0.58
**Procedure-related**				
Lateral wall acqu.	0.64	0.24	0.40–1.02	0.07
Inferior wall acqu.	0.90	0.24	0.56–1.43	0.66

**Abbreviations:** CI = confidence interval; PGY = post-graduate year;

## Data Availability

The study data are available upon request by contacting federico.cammertoni@policlinicogemelli.it.

## Abbreviations

The following abbreviations are used in this manuscript:

CABG	Coronary Artery Bypass Grafting
TTFM	Transit-time flow measurement
HRUS	High-resolution epicardial ultrasonography
MGF	Mean graft flow
PI	Pulsatility index
%BF	Percentage of backward flow
%DF	Percentage of diastolic filling
ACI	Acoustic coupling index
MAP	Mean arterial pressure
